# The Novelty Appraisal of the Feeling of Risk in Vehicles

**DOI:** 10.3390/ijerph192114259

**Published:** 2022-11-01

**Authors:** Meng Zhang, Meike Jipp, Klas Ihme

**Affiliations:** 1 Institute of Transportation Systems, German Aerospace Center (DLR), 12489 Berlin, Germany; 2 Institute of Transport Research, German Aerospace Center (DLR), 12489 Berlin, Germany; 3 Institute of Transportation Systems, German Aerospace Center (DLR), 38108 Braunschweig, Germany

**Keywords:** feeling of risk, novelty, facial expression, physiological responses, Component Process Model, in vehicles

## Abstract

Nowadays, vehicle assistance systems may assess the risks of the traffic situation with the help of advanced sensor technology and optimized algorithms. However, the passengers’ feelings of risk in the vehicle have been mostly neglected. According to the Component Process Model of emotions, during the feeling of risk, novelty is one of the relevant event appraisals leading to particular physiological and facial responses. In order to identify whether or not indicators for novelty appraisal may be used for detecting the feeling of risk of vehicle occupants, we investigated physiological responses and facial expressions of individuals experiencing the feeling of risk with different levels of novelty. This secondary analysis of an earlier simulator study revealed that pupil diameter amplitude, skin conductance level changes, and changes in and amplitude of activity in facial expressions (the inner and outer brow raiser, brow lowerer, upper lid raiser and lid tightener) were correlated with the reduction in the novelty, suggesting that they could indicate the novelty of the feeling of risk of vehicle occupants. Hence, this research provides evidence for the novelty appraisal of the feeling of risk. Furthermore, it informs research on affect-aware systems to identify and reduce the feeling of risk of vehicle occupants in order to help to keep trust in automated vehicles high.

## 1. Introduction

Risk and its assessment are essential concepts in the field of traffic safety. Particularly, in the last decade, research on autonomous driving has focused on different aspects of risks. Advanced sensor technology and optimized algorithms ensure that risks of the traffic situation are assessed and identified with increasing accuracy (e.g., [1]). However, drivers’ or passengers’ feelings of risk were barely focused on in studies related to automated driving. Summala ([2], p. 494) summarized the feeling of risk or fear experience as “an immediate reaction to a threatening stimulus, that involves the basic bodily mechanism”, which is distinguished from but associated with the objective risk, meaning the objective probability of being involved in an accident [3]. Much effort was devoted to identifying objective risks because automated driving functions (ADFs) are expected to reduce the accident rate compared to human drivers and ensure safety. However, identifying and reducing the feeling of risk of persons in vehicles could help to keep trust in ADFs high, for instance by providing drivers or passengers with ongoing feedback on the planned actions of the vehicle or by adapting its driving style [4,5].

Recognizing a person’s feeling of risk in vehicles is relevant for risk research. Particularly, in the context of acceptance of ADFs, the recognition of the feeling of risk in vehicles is an essential part. The risk of ADFs could refer to the system reliability relying on the individual knowledge of the system and the real-time individual judgment of traffic situations. The inconsistency between individual judgment and the state of the vehicle will lead to a decrease in system reliability. In a recent review [6], the feeling of risk was one of the most relevant impact factors of acceptance of ADFs, which suggested that an increase in the feeling of risk may reduce the trust in ADFs. In an ADF future, recognition of feelings of risk in vehicles and appropriately reacting to them may become a decisive factor for maintaining trust in ADFs and keeping passengers’ well-being high.

Previously, the feeling of risk or fear (from the point of view of emotional category) was studied as an emotional state, which belongs to and influences the decision-making loop [7,8] as well as driving behaviors [9,10]. According to the risk homeostasis theory [10,11], drivers continuously change their driving speed in order to maintain a certain level of the feeling of risk based on individual acceptance. Fuller [3] changed the feeling of risk into task difficulty in the task–capability interface model. Nevertheless, both theories have regarded the feeling of risk as a static state rather than a dynamic process. Hence, the consequences of the feeling of risk have received extensive attention. Previous studies focused on risk perception [12,13] and driving speed [14,15] under the influence of the feeling of risk, where the feeling of risk was mostly reported in the form of subjective reports. Given that detecting and mitigating feelings of risk in vehicles might be required in order to provide assistance to support vehicle occupants maintaining trust in ADFs, reliable measurements with consideration of the dynamic nature of emotion need to be investigated.

According to a component process definition of emotion [16], emotional processes are associated with the following components: cognitive component (appraisal), neurophysiological component (e.g., physiological responses), motivational component (action tendencies), motor expression component (e.g., facial and vocal expression) and subjective feeling component. The subjective reports mentioned above are measurements of a subjective feeling component. The measurements of neurophysiological and motor expression components are widely implemented in in-vehicle studies. Electrodermal activity (EDA) and electrocardiography (ECG) are measurements of physiological responses and have frequently been applied to recognize emotion in vehicles [17,18,19]. In particular, skin conductance level (SCL), one of the indicators of autonomic nervous system activity, has been described to represent drivers’ arousal when experiencing fear [9]. Furthermore, in a recent simulator study [20], pupil dilation was suggested as an indicator of perceived risk in vehicles. Through video recognition based on the Facial Action Coding System (FACS [21,22]) and facial electromyography (EMG), facial expressions were also often applied to recognize emotion in vehicles [23,24,25]. However, whether there are universal expressive patterns for certain emotions is still debated. Other researchers based their approaches to assessing emotions on the theoretical basis of dimensional emotion models [18,26,27], mostly considering arousal in the framework of the arousal–valence dimensional model [28,29]. More recently, in addition to valence and arousal, more dimensions (e.g., novelty) were suggested to describe the full space of emotional experience [30,31]. In this vein, a recent study suggested understanding facial expressions with regard to the time courses of and the mechanisms underlying the generation of emotional appraisals [32].

The Component Process Model (CPM) reveals the underlying mechanisms of emotion processes constructing the five components mentioned above [33,34]. In the assumption of the CPM, the appraisal of an event triggers changes in action tendencies, autonomic nervous system (ANS) and somatic nervous system (SNS), which are then integrated and represented as individual feelings [35]. Thus, the feeling of risk in vehicles, according to the CPM, could be understood as an emotion process of fear: an individual evaluation of a threatening traffic event leads to a series of changes in actions, physiological responses (e.g., increase in heart rate) and motor expressions (e.g., facial expressions), which is interpreted as the feeling of risk by an individual. Hence, this evaluation can be considered as a multidimensional process. The following aspects would be sequentially appraised: Is the event unfamiliar (*novelty*)? Is it unpleasant (*pleasantness*)? Does it hinder the individual goal (*goal significance*)? Can it be coped with (*coping potential/power*)? Previous studies, in which appraisal components were manipulated, supported the CPM from the perspective of action tendencies [36], ANS [37] and SNS [38]. Studies on action tendencies [36] found that pleasantness or unpleasantness appraisals could lead to approach (e.g., step forward) or avoidance (e.g., step backward) behaviors. Reisenzein et al. [37] reviewed that novelty appraisals of stimuli were supposed to drive several changes in the ANS including increased skin conductance as well as decreases in heart rate and pupil dilation. With regard to SNS, Scherer et al. [38] integrate empirical evidence to determine the relationship between action units (AUs, atomic units of facial action) and appraisal components assuming that the occurrence of a facial expression is a sequential cumulative process, which is triggered by appraisal components in sequence. Scherer et al. [38] predicted that novelty appraisal in fear would trigger the inner and outer brow raiser (AUs 1 and 2), brow lowerer (AU 4), upper lid raiser (AU 5), lid tightener (AU 7), jaw drop (AU 26) and nostril dilator (AU 38).

Only a few studies considered the framework of the CPM and the appraisal theory in the investigation of feelings of risk in vehicles. Besides the frequency and the consequences of emotions, Mesken [17] suggested the investigation from the perspective of personal characteristics of the occupants and traffic events, meaning that understanding how occupants appraise the traffic events could be relevant as well. Furthermore, Mesken [39] also suggested multimodal measurements of emotion in vehicles, which is consistent with the multicomponent assumption of CPM. An on-road study [17] showed the difference in heart rate between emotions that were provoked by the appraisal of the blamed party in traffic events. Anxiety, a general type of fear [40], was considered as the result of the situation, i.e., blame, associated with increased heart rate [17]. This result is consistent with the result from a questionnaire study [41]. In a driving simulator study [32], facial expressions based on the FACS were used to successfully indicate the appraisal of high novelty and low power in fearful traffic situations. However, reliable multimodal measurements of the feeling of risk in vehicles with consideration of the individual appraisal of the situation still need more empirical validation.

The aim of this work is to validate multimodal measurements of the feeling of risk in vehicles considering the framework of the CPM and to provide evidence for the novelty appraisal related to it. Therefore, we studied the impact of novelty on drivers’ physiological responses and facial behavior by repeatedly presenting threatening traffic stimuli. Here we executed a secondary analysis of a previous driving simulator study [42]. We studied the correlation of the repetition of three traffic events with drivers’ physiological responses and with facial behaviors; it was assumed, according to Reisenzein et al. [43], that increasing familiarity with events will lead to a reduction in novelty.

Physiological responses play an essential role in emotion measurements. As mentioned previously, several physiological responses, such as increased skin conductance as well as decreases in heart rate and pupil dilation, were reported to be associated with novelty appraisals [37]. In line with previous research, we hypothesized that physiological responses are correlated with the reduction in the novelty of events.

Facial expressions as assessed with video observations are one of the commonly used in-vehicle measurements of emotion [23,24,25] because the assessment is contactless and unobtrusive. As mentioned previously, Scherer et al. [38] predicted that AUs 1, 2, 4, 5, 7, 26 and 38 could be associated with novelty appraisal in fear. Thus, we hypothesized that these AUs’ activities are correlated with the reduction in the novelty of events in the vehicle context.

## 2. Methods

In order to evaluate the hypotheses, we reanalyzed data from an earlier study [42]. The original study intended to induce two target emotional states (fear and neutral) with differences in the emotional dimension of power through threatening and challenging traffic events in a within-participants design. The participants were asked to drive in four urban driving scenarios (two for each target emotional state). As a cover story, participants had the task of delivering a parcel within seven minutes. After the entire experiment, the true purpose of the study was revealed. The scenario was accomplished in a driving simulator consisting of three screens and a steering wheel as well as gas and brake pedals to control a virtual car in a driving simulation (Virtual Test Drive, Vires, Germany). The participants were asked to complete a demographic questionnaire before the experiment started and a subjective rating questionnaire after each scenario. According to the employed subjective rating questionnaire Positive And Negative Affect Schedule (PANAS, original: [44]; German version: [45]), the item “scared” had a significantly elevated score in the fear scenario (*M* = 2.61, *SD* = 0.98, *Z* = 3.54, *p* < 0.001 *r* = 0.84). Additionally, the participants’ rating on novelty level was also significantly elevated (*M* = 4.56, *SD* = 1.95, *Z* = 3.63, *p* < 0.001, *r* = 0.85). The subjective rating suggested that the induction of fear was successful and that the novelty appraisal appeared as intended.

### 2.1. Participants

Eighteen participants (four females) ranging from 22 to 40 years old (mean age = 27.5 years, standard deviation = 4.5 years) took part in the study. All participants possessed a valid driving license and had at least two years of driving experience. Before the start of the study, participants were informed about the video recording, potential risks of driving in simulators (e.g., the experience of simulator sickness) according to the simulator safety concept and the rough duration of the experiment. The participants were informed that they could take a break or abort their participation at any time. All the participants provided written informed consent to part in the study and the video recording. As reimbursement for their time, the participants received EUR 10 per commenced hour for their participation. After finishing, the participants were informed about the true goal of the experiment (evoking certain emotions) and the necessity to conceal this goal with a cover story.

### 2.2. Materials and Procedure

**Figure 1:**
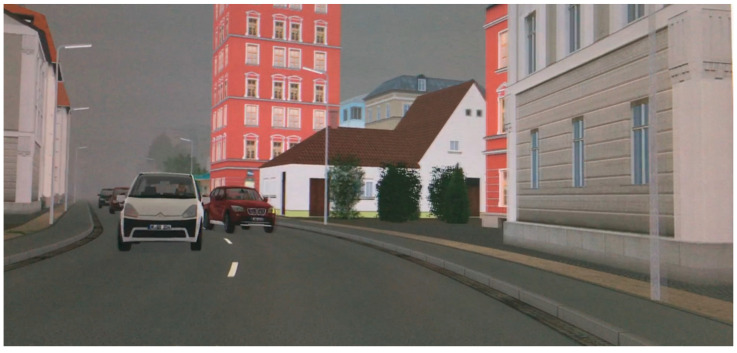
A screenshot of the driving simulation during one of three threatening traffic events in the fear scenario, where participants were faced with a car swerving into the ego lane during an overtaking maneuver.

In this secondary analysis, we focused on one of the fear scenarios, in which the participants entirely experienced the three events. The scenario had a length of ~5 km (3.1 miles) and started with a one-minute drive without any events. Afterward, three threatening traffic events, a crash or almost-crash produced by a vehicle swerving abruptly from the opposite lane (see Figure 1), occurred with an inter-event interval of about one minute (depending on the driving speed). We regarded the occurrence of the swerving vehicle as the onset of each event and extracted the epoch from one second before to ten seconds after the event onset for the event-related analysis.

### 2.3. Measurements

**Figure 2:**
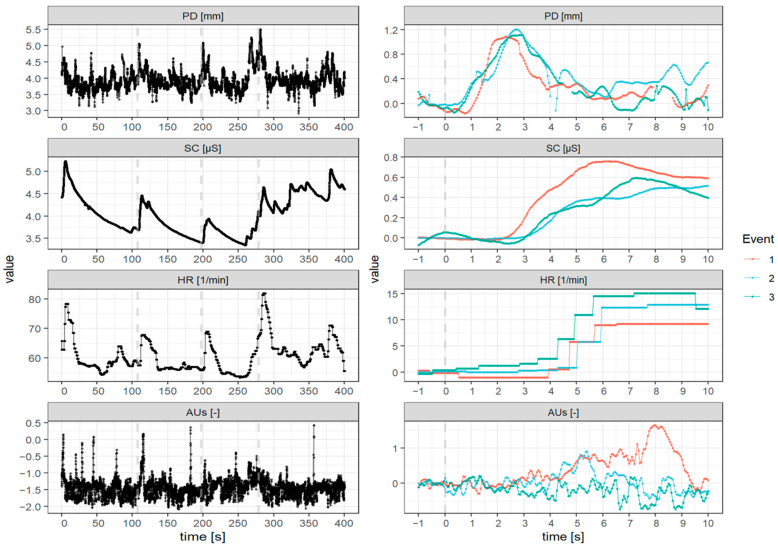
Pupil diameter (PD), skin conductance (SC), heart rate (HR) and action units (AUs) of one participant in the simulator drive with three threatening traffic events (onset of events in dashed lines). Left: raw data for the entire drive; right: event-logged data after baseline subtraction.

Pupil dilation, EDA, ECG and the participants’ facial expressions were recorded during the entire experiment. In the following, the measurement and processing of the signals and the extraction of relevant parameters are described (see Figure 2). In order to reduce the difference between the participants, all changes in parameters were scaled within every drive and adjusted, whereby the average value in the one second before event onset of the respective participant and event was subtracted. Note that the averaged value in the following section refers to the linear average across the value in a certain time interval for each event and each participant. For instance, the averaged pupil diameter of participant x from event 1 onset to ten seconds refers to the linear averaged value of the red line from 0 to 10 in the top right panel of Figure 2.

#### 2.3.1. Pupil Dilation

A SmartEye-Pro system (SmartEye, Gothenburg, Sweden) with two cameras, which were mounted on both sides of the dashboard, was used to track and record pupil diameter (PD) with a sampling rate of 120 Hz. SmartEye-Pro enables quantifying the measurement quality of PD, according to which raw data obtained with a quality lower than 0.5 were excluded and extreme PD values (below 1 mm or above 9 mm) were removed. As a result, the PD data of four participants and four scenarios, in which above 80% of data points had to be removed, were excluded. On average, 71% of the data points remained in the rest of the participants and scenarios. Additionally, the gaps in the remaining data were interpolated by natural splines. Our analyses focused on the following parameters:

- PD changes, quantified as the averaged pupil diameter from event onset to ten seconds after the event subtracted from a baseline (= the mean of the last second before event onset).
- PD amplitude, quantified as the maximum of PD after event onset subtracted from a baseline (= the mean of the last second before event onset).

#### 2.3.2. EDA

We employed a finger sensor (Heally, SpaceBit, Eberswalde, Germany) on the forefinger of the non-dominant hand to assess skin conductance with a sampling rate of 25 Hz. The package NeuroKit [46] (version: 0.1.5) for the programming language Python was used to process the raw skin conductance signal (SC), whereby the signal was decomposed into tonic (skin conductance level: SCL) and phasic components (skin conductance responses: SCRs). Both indices were assumed to reflect sympathetic neuronal activity [47]. The following parameters were relevant for the further analyses:

- SCL changes, quantified as the averaged SCL from event onset to ten seconds after subtracted from the baseline (= the average of the last second before event onset).
- SCR amplitude, quantified as the amplitude of the first SCR after event onset.
- NSCR, which is the number of SCRs in the ten seconds after event onset.

#### 2.3.3. ECG

ECG was measured with three electrodes placed on the participant’s chest and recorded by a Heally device with a sampling rate of 500 Hz. We used the package NeuroKit [46] (version: 0.1.5) for the programming language Python to process the data, including artifact elimination (based on the algorithm of Lipponen and Tarvainen [48]), R-peak extraction and heart rate (HR) calculation. The relevant parameters involved the HR and the heart rate variability:

- HR changes, quantified as the averaged HR from event onset to ten seconds after subtracted from a baseline (= the mean of the last second before event onset).
- HR amplitude, quantified as the maximum of the HR in the ten seconds after event onset.
- RMSSD, quantified as the square root of the mean of the sum of successive differences between adjacent RR intervals in the ten seconds after event onset.

#### 2.3.4. Facial Behaviors

The participants’ faces were recorded from the front with a network camera (Abus, Wetter, Germany) with a frame rate of 15 frames per second and a resolution of 1280 × 720 pixels. According to the FACS, the activity of facial expressions can be described based on activity in atomic units of facial action, the AUs. We used the Attention Tool FACET Module (FACET, iMotions, Singapore) to quantify the frame-to-frame activity of the facial AUs. Nineteen AUs were encoded in terms of the intensity of the activation. Each of them was assigned a numerical value. As mentioned above, the AUs 1, 2, 4, 5 and 7 (see details in Table 1) were assumed to associate with the appraisal component of novelty. The compound (linear average) of upper facial AUs 1, 2, 4, 5 and 7 was regarded as the indicator of the appraisal component of novelty. Jaw drop (AU 26) was not considered, because lower facial muscles were found to be inhibited after a novelty stimulus [49]. Additionally, nostril dilator (AU 38) was not used because it was not covered by the software package. The following parameters were relevant for the further analysis:

- AU changes, quantified as the averaged AUs of novelty from event onset to ten seconds after subtracted from a baseline (= the mean of the last second before event onset).
- AU amplitude, quantified as the maximum of AUs of novelty after event onset subtracted from a baseline (= the mean of the last second before event onset).

### 2.4. Statistical Analyses

In order to reveal the correlations between novelty and the extracted parameters of physiological responses and facial behavior across individuals, we employed the Repeated Measures Correlation (Rmcorr) as implemented in the package rmcorr [50] (version: 0.4.5) for the R programming language. There were three threatening traffic events for each scenario; we used an event index (1, 2 and 3) to represent the event order. The correlation between the event index and each of the physiological response parameters and the correlation between the event index and facial behavior parameters were analyzed by Rmcorr. The correlation coefficient (*r*), degree of freedom (*df*), confidence interval (*CI*) and *p*-value (*p*) were provided and reported. Furthermore, the mean (*M*) and standard deviation (*SD*) of parameters in each event were also included. A significance level of *α* = 0.05 was used for all tests.

### 2.5. Explorative Analysis of Peak Time

**Figure 3:**
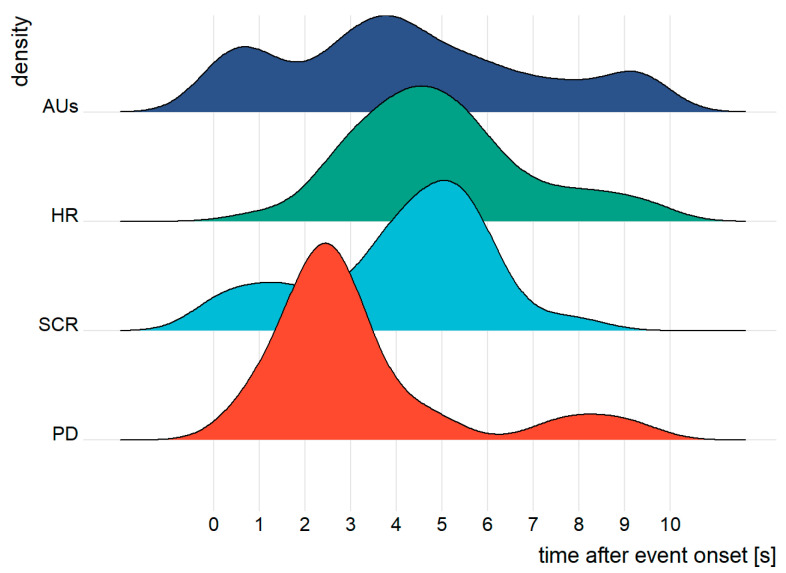
Distributions of peak times of PD (red), SCR (light blue), HR (green) and novelty AUs (dark blue) after event onset.

In an additional explorative analysis, we investigated the peak time of PD, SCR, HR and the AUs (see Figure 3). According to the results of Shapiro–Wilk normality tests, the peak times were not normally distributed (*W* = 0.97, *p* < 0.01). We employed Scheirer–Ray–Hare tests assuming that PD, SCR, HR and AUs had different peak times and that they could be impacted by the event index, namely the different novelty levels. The Scheirer–Ray–Hare test was performed in the package rcompanion [51] (version: 2.3.26) for the R programming language (version: 3.6.3). We considered the time point of the maximum of PD, SCR, HR and AUs in the ten seconds after event onset as the peak time of corresponding parameters. We excluded the peak times of zero and ten assuming that there were no local maximum values in those cases. The results were presented as Z-score (*Z*), *df* and *p*. Additionally, *M*, *SD* and *CI* were reported.

## 3. Results

### 3.1. Physiological Responses

#### 3.1.1. Pupil

According to Rmcorr, PD amplitude and the event index showed a negative correlation (see Table 2) suggesting that the PD amplitude was decreasing with decreasing novelty, which is in line with the hypothesis that physiological responses are correlated with the reduction in the novelty of events.

#### 3.1.2. EDA

According to Rmcorr, SCL changes and the event index showed a negative correlation (see Table 2), which is in line with the hypothesis that the SCL was decreasing with decreasing novelty. However, SCR amplitude and NSCR showed no correlation with the event index (see Table 2).

#### 3.1.3. ECG

According to Rmcorr, HR changes and amplitude as well as RMSSD showed no correlation with the event index (see Table 2). These results differ from the hypothesis.

### 3.2. Facial Behaviors

According to Rmcorr, both AU changes and amplitude showed a negative correlation with the event index (see Table 2), which is in line with the hypothesis that both parameters were decreasing with decreasing novelty.

### 3.3. Peak Time

Scheirer–Ray–Hare test revealed that there was a significant main effect of the parameters on the peak time, suggesting that PD, SCR, HR and AUs may have their maximum at different time points (*Z* = 3.84, *p* < 0.01). However, neither the event index (*Z* = 0.42, *p* = 0.68) nor the interaction of parameter and event index (*Z* = 0.56, *p* = 0.58) showed significant effects. Specifically, PD reached the peak at 3.18 (*SD* = 2.11 *CI* = (2.53, 3.83)) s after event onset. SCR reached the peak at 4.08 (*SD* = 1.90, *CI* = (3.51, 4.64)) s. HR reached the peak at 4.96 (*SD* = 1.93, *CI* = (4.40, 5.92)) s. AUs reached the peak at 4.25 (*SD* = 2.78, *CI* = (3.49, 5.01)) s (see Figure 3).

## 4. Discussion

The goal of this study was to investigate whether or not physiological responses and facial behavior could be an indicator of the novelty appraisal of the feeling of risk of vehicle occupants as well as to validate multimodal measurements of the feeling of risk in vehicles considering the framework of the CPM. Through a secondary analysis of our previous driving simulator study, in which the feeling of risk was induced by three threatening traffic events, we revealed that physiological responses and facial behavior were significantly correlated with the event order. Our results suggest that PD amplitude, SCL changes, and changes in and amplitude of certain AUs (AUs 1, 2, 4, 5 and 7) indicate the novelty of feeling of risk in vehicles by showing a negative correlation with the reduction in the novelty of events. On the one hand, the findings of the study provided consistent evidence of the correlation between novelty appraisal and physiological responses as well as facial behavior. On the other hand, the study provided a new perspective for ADFs on measuring the feeling of risk in vehicles with consideration of the underlying mechanisms of emotions.

In this work, PD, EDA and ECG were considered as measurements of physiological responses. PD amplitude and SCL changes decreased along with the reduction in the novelty of events, suggesting the association with the novelty appraisal, which is consistent with the result from a previous study [37]. However, not all the parameters of the physiological responses showed an association with the novelty appraisal: PD changes showed a closer to zero correlation coefficient compared with PD amplitude. This result implies that the duration of pupil dilation may be short-lived; thus, temporal parameters such as PD amplitude might be more sensitive to the novelty in the feeling of risk. With regard to EDA, SCR amplitude did not correlate with the reduction in the novelty of the events either. The same pattern of no correlation was observed for all ECG parameters. These results differ from previous research [49,52]. However, the specificity of these parameters as indicators of novelty is still under debate. Notably, PD and SCL changes were also used to indicate emotional arousal [53,54], and HR was used to indicate valence [55] in earlier work. In our study design, arousal and valence were not manipulated. Therefore, the specificity of the mentioned physiological response parameters as indicators of novelty should be investigated in further studies considering a manipulation of other appraisals or emotional dimensions.

The compound of facial AUs 1, 2, 4, 5 and 7 was used to indicate the appraisal component of novelty. The AU changes and amplitude decreased along with the reduction in the novelty of events, suggesting that both parameters could be considered as indicators of novelty appraisal in the feeling of risk. The result is consistent with the prediction of Scherer et al. [38]. It is in line with the findings in EMG studies: In a previous EMG study, facial muscle activity in the frontalis region [56] corresponding to AUs 1 and 2 as well as around eyelids (orbicularis oculi) [49] corresponding to AUs 6 (cheek raiser) and 7 was revealed to be related with the appraisal component of novelty. Similarly, here, it has to be noted that the specificity of facial expression of emotions is still under debate [57]. Particularly, one study reported that people hardly show one or several consistent facial expressions after novelty or unexcepted stimuli [43]. Thus, the current study integrated the novelty-related facial AUs without expecting them to activate simultaneously.

In the explorative analysis, we additionally investigated the peak time of PD, SCR, HR and AUs. In contrast with the aforementioned physiological responses and facial expression parameters, their peak time was not impacted by the event order, corresponding to the novelty level. However, the results showed that PD, SCR, HR and AUs reached the peak at different time slots: PD’s peak was the earliest one among the parameters, and it was between 2.53 s and 3.83 s after event onset. SCR reached the peak in the time between 3.51 s and 4.64 s after event onset, followed by AUs and HR. Note that the larger SD and the wide distribution (see Figure 3) of AUs might imply that individual differences impacted the peak time of AUs. Given that different channels may have different peak times, our study may provide a new perspective for measuring feelings of risk in vehicles. For instance, with regard to multimodal measurements, the dynamic nature of the emotion process, particularly the specific activation time of each channel, should be considered in order to optimize the measurement.

In order to increase the immersion of participants, the experiment was conducted under the framework of a manual driving simulator with three screens. The rating of “scared” (*M* = 2.61, *SD* = 0.98) on a Likert scale from 1 to 5 and novelty (*M* = 4.56, *SD* = 1.95) on a Likert scale from 1 to 9 suggested that fear was induced and novelty appraisal appeared. However, the ratings showed only a moderate level of “scared” and novelty. It could be interpreted that the induced feeling of risk may be limited by the safety feeling in the virtual environment. Hence, the feeling of risk induced in a simulator environment may deviate from the one triggered by spontaneous events in real traffic environments. With regard to the generality, the framework of automated driving should also be considered in future research. Furthermore, as mentioned previously, the main limitation of the specification of the indicators of novelty was not proven. PD amplitude and SCL changes decreased along with the event order, which may also be interpreted as the effect of a reduction in arousal, because both parameters were proven to be associated with sympathetic activity [54]. Assuming that emotions could be displayed in a multidimensional space, the dimensions besides novelty (e.g., arousal and valence) could be manipulated by inducing more emotions in future research (e.g., stress was considered as an emotional/cognitive process with similar arousal and valence level as fear, but lower novelty [30]). Another limitation is the rather small size of the sample (*N* = 18). Small sample sizes could reduce the power of the results of correlation analyses [50,58]. A validation of the study results with a larger sample size is therefore desired. Additionally, small sample sizes hindered the research possibilities on demographics. Previous studies showed that gender [59] and driving experience [20] may impact event appraisals and corresponding responses. Hence, future studies should investigate the effect of gender and experience on novelty appraisal in the feeling of risk with larger participant samples.

The direction for improvement after the feeling of risk in vehicles is measured is still one of the topics for discussion in the application field. As mentioned in the introduction, trust in ADFs would be influenced when the occupants experience risk, even though the situation is not critical. At this point, reacting to the feeling of risk through the adjustments in driving style may help occupants to regain their trust. Thus, the applicational study on reduction in the feeling of risk in vehicles could be considered.

## 5. Conclusions

In this study, the multimodal measurements of the feeling of risk in vehicles considering the framework of the CPM were validated. According to the accomplished secondary analysis of an earlier driving simulator study, physiological responses (PD amplitude, SCL changes) and facial expressions (changes in and amplitude of AUs 1, 2, 4, 5 and 7) were correlated with the reduction in the novelty. This suggests that these parameters could be considered as indicators of novelty when assessing the feeling of risk of vehicle occupants. Additionally, the peak times of PD, SCR, HR and AUs were tentatively investigated. They reached the peak at different time slots, and PD’s peak was the earliest one among the parameters. In sum, the results of this research provide evidence for the appraisal of novelty in emotion. Furthermore, our work can inform the design of affect-aware systems to identify and later mitigate feelings of risk of vehicle occupants as a basis for keeping trust in ADFs high.

## Figures and Tables

**Table 1:** Description of facial action units (AUs) used in the study.

AUs	Description
1	inner brow raiser
2	outer brow raiser
4	brow lowerer
5	upper lid raiser
7	lid tightener

**Table 2:** Correlation of aggregated parameters and events sorted by correlation coefficient (*r*).

Parameter	*r*	*df*	*CI*	*p*	*M* ± *SD* in Event		
					Event 1	Event 2	Event 3
AU changes	−0.52	35	(−0.727, −0.226)	0.001 ***	0.06 ± 0.4	−0.06 ± 0.23	−0.21 ± 0.27
PD amplitude (mm)	−0.399	23	(−0.697, 0.015)	0.048 *	1.14 ± 0.72	1.21 ± 0.36	0.61 ± 0.37
SCL changes (µS)	−0.393	35	(−0.642, −0.069)	0.016 *	0.26 ± 0.29	0.18 ± 0.19	0.14 ± 0.2
AU amplitude	−0.362	35	(−0.62, −0.032)	0.028 *	0.72 ± 0.96	0.52 ± 0.23	0.31 ± 0.16
HR changes	−0.319	35	(−0.589, 0.016)	0.055	6.62 ± 3.81	6.61 ± 4.93	4.02 ± 5.29
PD changes (mm)	−0.282	23	(−0.622, 0.148)	0.172	0.28 ± 0.31	0.42 ± 0.26	0.02 ± 0.35
HR amplitude	−0.174	35	(−0.48, 0.169)	0.302	12.69 ± 5.07	13.17 ± 5.97	10.85 ± 7.23
SCR amplitude (µS)	−0.109	32	(−0.441, 0.249)	0.54	0.27 ± 0.33	0.73 ± 1.57	0.3 ± 0.35
RMSSD	−0.094	35	(−0.414, 0.247)	0.582	35.92 ± 18.84	40.68 ± 25.32	32.82 ± 22.5
NSCR	0.032	35	(−0.305, 0.361)	0.851	1.44 ± 1.1	1.5 ± 1.04	1.5 ± 0.99

* *p* < 0.05; *** *p* < 0.001.

## Data Availability

Not applicable.
